# Rapid identification of medicinal plants via visual feature-based deep learning

**DOI:** 10.1186/s13007-024-01202-6

**Published:** 2024-05-31

**Authors:** Chaoqun Tan, Long Tian, Chunjie Wu, Ke Li

**Affiliations:** 1 College of Intelligent Medicine, Chengdu University of Traditional Chinese Medicine, Chengdu, 611137 China; 2 School of Electronic Engineering and Computer Science, Queen Mary University of London, London, E1 4NS UK; 3 National Key Laboratory of Fundamental Science on Synthetic Vision, College of Computer Science, Sichuan University, Chengdu, 610065 China; 4 Innovative Institute of Chinese Medicine and Pharmacy/Academy for Interdiscipline, Chengdu Univesity of Traditional Chinese Medicine, Chengdu, China

**Keywords:** Medicinal plants, Identification, Deep learning, Image recognition, Masked autoencoders

## Abstract

**Background:**

Traditional Chinese Medicinal Plants (CMPs) hold a significant and core status for the healthcare system and cultural heritage in China. It has been practiced and refined with a history of exceeding thousands of years for health-protective affection and clinical treatment in China. It plays an indispensable role in the traditional health landscape and modern medical care. It is important to accurately identify CMPs for avoiding the affected clinical safety and medication efficacy by the different processed conditions and cultivation environment confusion.

**Results:**

In this study, we utilize a self-developed device to obtain high-resolution data. Furthermore, we constructed a visual multi-varieties CMPs image dataset. Firstly, a random local data enhancement preprocessing method is proposed to enrich the feature representation for imbalanced data by random cropping and random shadowing. Then, a novel hybrid supervised pre-training network is proposed to expand the integration of global features within Masked Autoencoders (MAE) by incorporating a parallel classification branch. It can effectively enhance the feature capture capabilities by integrating global features and local details. Besides, the newly designed losses are proposed to strengthen the training efficiency and improve the learning capacity, based on reconstruction loss and classification loss.

**Conclusions:**

Extensive experiments are performed on our dataset as well as the public dataset. Experimental results demonstrate that our method achieves the best performance among the state-of-the-art methods, highlighting the advantages of efficient implementation of plant technology and having good prospects for real-world applications.

## Introduction

Chinese Medicinal Plants (CMPs) can be directly used in the clinical practice of traditional Chinese medicines. It has been an essential part of healthcare for thousands of years, with a focus on using natural plant-based remedies to promote health, prevent illness, and treat various medical conditions [1–3]. CMPs are employed as either a primary or complementary method to address a diverse spectrum of health concerns, spanning from minor ailments to chronic conditions. The important role of CMPs in the prevention and treatment of many epidemic, chronic, and infectious diseases, such as COVID-19, CMPs has been widely demonstrated and recognized by the international community [4–6]. The quality of CMPs is one of the major factors in ensuring medication safety and clinical security [7–10].

Typically, biological techniques and chemical methods, such as mass spectrometry, gas chromatography, etc., can be used for adulteration detection [11–13]. However, these analyses require highly trained professionals, and also it is time-consuming. On the other hand, molecular markers serve as a fast and promising analytical way, but it is cumbersome and high professional threshold [14, 15]. Additionally, the evaluation of CMPs by manual identification lacks objectivity and scientificity. As an effective alternative, the research hot spot for the identification of CMPs based on intelligent sensory technology (such as electronic nose, electronic tongue, and electronic eyes) has aroused strong attention. Previous works [16–18] have demonstrated the effectiveness of discrimination, however, those require expensive equipment and are not efficient. Additionally, image processing by hand-designed features relies heavily on the analysis of shallow visual features, lacking the capture of high-level semantic features. Consequently, the approaches for rapid and accurate detection of CMPs are necessary for practical use and market demands.

With the continuous innovation and research in computer technology, deep learning in following-up on the effects of image processing has been widely recognized for the identification of food, plant, agriculture, medical care, and multiple fields [19–23]. It has also been used for the identification of CMPs. Zhou et al. [24] combined near-infrared spectroscopy and convolutional neural networks to analyze medicinal plants from different origins. Wang et al. [25] proposed hyperspectral imaging assisted by an attention mechanism and a long short-term memory network to identify the origin of the coix seed and predict the nutritional content. Miao et al. [26] fused ConvNeXt with the ACMix network to extract features and classify traditional Chinese medicine. Bai et al. [27] combined deep learning and spectral fingerprint features to accurately predict the soluble solids content of jujube in multiple geographical areas. Yan et al. [28] used visible/near-infrared combined with deep learning to identify the geographical origin of licorice. Yue et al. [29] employed near-infrared 2DCOS images combined with a residual neural network to identify the origin of Yunnan’s big leaves. Compared with widely used generative adversarial networks (GANs) [30, 31] and CNN-based methods [32–34], the Masked AutoEncoders (MAE) [35] have caused public concerns due to reducing dependence on data. In this paper, our goal is to investigate a rapid and effective strategy for identifying the different varieties of CMPs. Inspired by MAE and CoAtNet, a hybrid structure by fusing MBConv [36] and Transformer [37] has been designed to better obtain the local details and global features for the classification of CMPs.

**Fig. 1:**
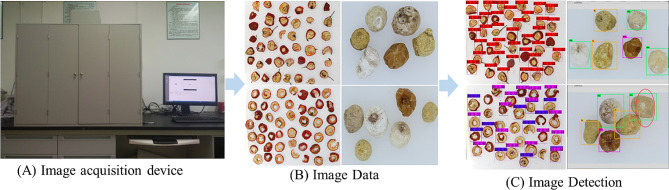
The image detection to detection results. (**A**) is the image acquisition device. The device is composed of a box, a light system, and an image acquisition system, which can provide stable and consistent environmental conditions. (**B**) is the obtained medicinal plant images of different types. (**C**) is the detected images with bounding boxes

To the best of our knowledge, there is no public medicinal fruit plants dataset, thus, we create a new dataset. We create a comprehensive visual multi-varieties CPMs images dataset, where high-resolution images are captured using a self-developed acquisition device, the details are shown in Sect. 2. On the other hand, to enhance MAE for extracting global features and reducing information loss, we propose a novel framework. The overview of our model is illustrated in Fig. 1, and details of our proposed methods can be found in Sect. 3. Finally, the experimental results and analysis are shown in Sect. 4, with a conclusion drawn in Sect. 5. The contributions of this study are highlighted as follows:

(1) Utilizing self-developed equipment to acquire our dataset, which is the first publicly dataset related to medicinal fruit plants.

(2) Compared with the previous works, the proposed method addresses the limitations of MAE in extracting global features and reduces information loss. By combining a new pre-training paradigm integrating self-supervised and supervised label information, it can mitigate the model overfitting to imbalanced data and enhance adaptability.

(3) In response to the characteristics of the dataset, a novel random data augmentation method is proposed to enhance the model’s focus on edge regions and feature extraction by randomly adding shadows to local areas.

(4) Extensive experiments are performed on our dataset as well as public datasets. The experimental results show that our model achieves the highest accuracy among state-of-the-art models. Our proposed model has excellent practical value for plant technology.

## Materials

### Sample preparation

All samples are obtained from the Lotus Pond medicinal market in Chengdu. Our collection has 14 different types of samples as long as their derived products. These samples are certified by experts from the Chengdu Institute of Food and Drug Control (Chengdu, China). The dry samples are derived from intact samples and are stored in ordinary cold storage.

### Data acquisition

**Fig. 2:**
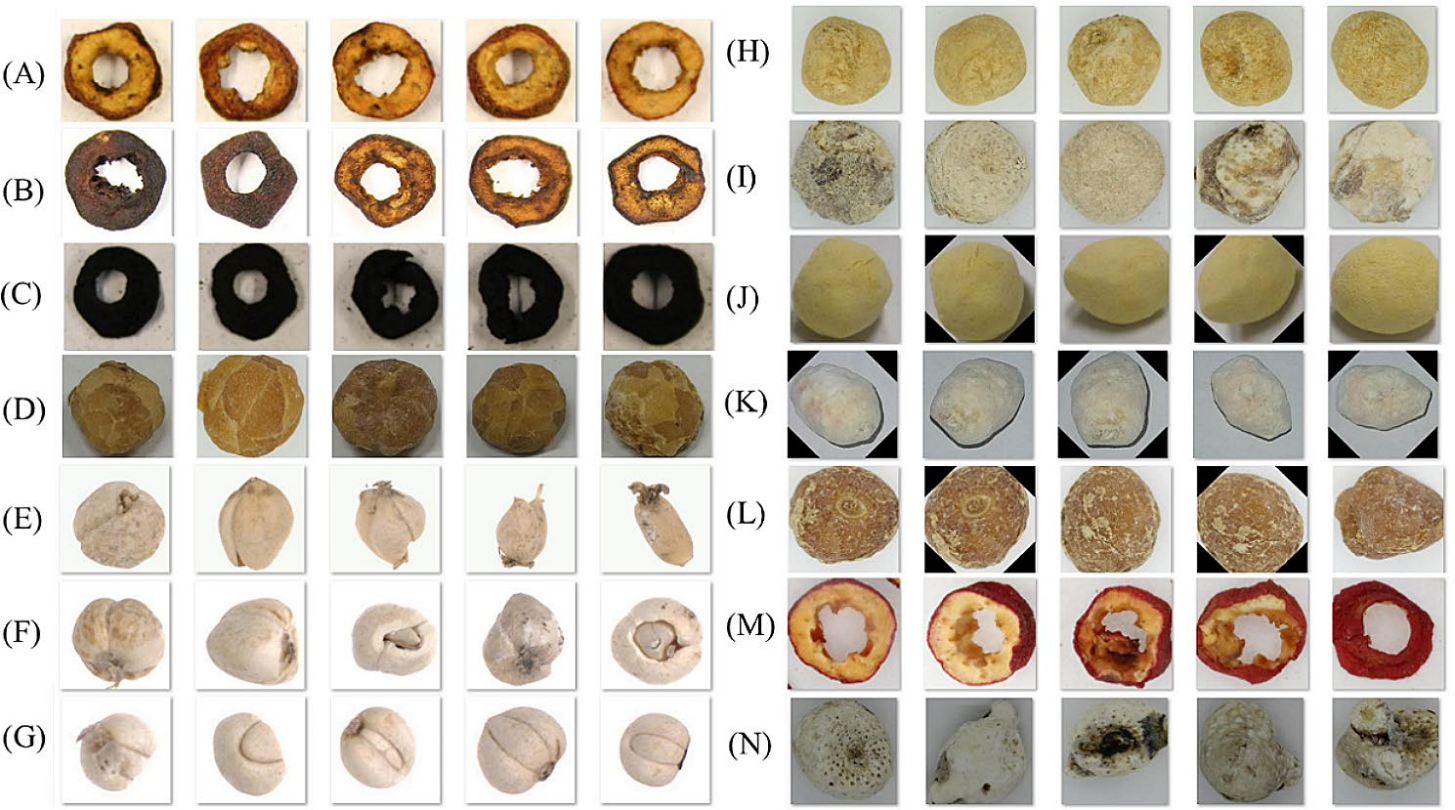
The dataset consists of 14 different CHMs and their produced products. Namely (**A**) *chaoshanzha* (**B**) *jiaoshanzha* (**C**) *shanzhatan* (**D**) *jiangbanxia* (**E**) *lubei* (**F**) *qingbei* (**G**) *songbei* (**H**) *fabanxia* (**I**) *shengbanxia* (**J**) *jingbanxia* (**K**) *shuibanxia* (**L**) *jiangnanxing* (**M**) *shanzha* (**N**) *qingbanxia*

A self-developed high-resolution data acquisition device (Canon EOS 60D) is used to acquire images as shown in Fig. 2A. The device is composed of a box, a light system, and an image acquisition system, which can provide stable and consistent environmental conditions. The image acquisition process is illustrated in Fig. 2.

**Fig. 3:**
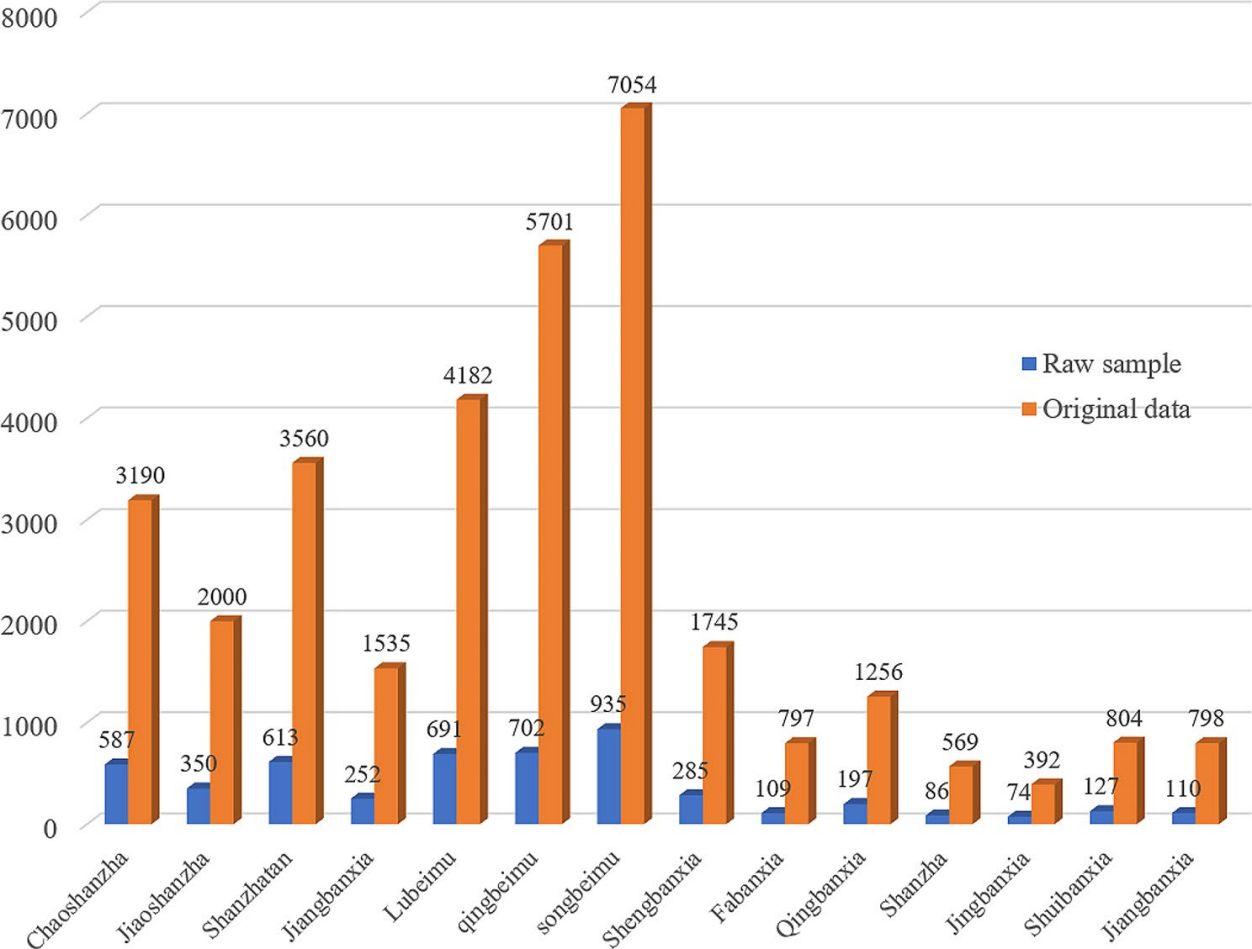
The distribution of the number of images within each CMP in our dataset. The blue represents the raw samples, while the orange is the collected original data

The box is made of wood and has a reflective gray coating with a reflectivity of 18%. PHILIPS Graphical TL-D light with a temperature of 5000 K is used in the light system. Four light tubes and scattering plates are utilized to eliminate any shadowing during the image-capturing process. All images are captured using a 35 mm CMOS sensor with a resolution of 5120 × 3840, as shown in Fig. 2B. Images are annotated and cropped to obtain a target. (Fig. 2C), while incomplete, blurry, and inappropriate images are removed. Our dataset is shown in Fig. 3.

*shanzha* is a medicinal and edible plant, which commonly applied in clinical practice by slices. In our dataset, there are four varieties from the same origin, including *shanzha*, *chaoshanzha*, *jiaoshanzha*, and *shanzhatan*. They are fired at different temperatures by sliced *shanzha*. For example, *chaoshanzha* is fired at 100℃, *jiaoshanzha* is fired at 150℃, and *shanzhatan* is fired at 200℃. With the fluctuation of temperature during frying, there are alterations in both the morphology and color, leading to variations in pharmacological effects. Similarly, *jiangbanxia*, *fabanxia*, *qingbanxia*, and *jingbanxia* are from the same origins, while they are obtained from mature harvested *banxia* by different processing methods. Specifically, *qingbanxia* is obtained by purifying *banxia*, *jiangbanxia* is made by mixing ginger juice and *banxia*, and *fabanxia* is obtained by soaking *banxia* in licorice lime liquid. Additionally, *jingbanxia* is a highly valuable medicinal plant prepared by mixing *banxia* with various adjuvants. *jiangnanxing* is a processed product derived product from *Tiger’s Paw Southern Star* and has completely different medicinal effects from *banxia*. On the other hand, *shuibanxia* has a different origin and effects from *banxia*. Furthermore, *lubeimu*, *qingbeimu*, and *songbiemu* are three different species of *chuanBeimu*, they have different market values due to their different morphology and color.

**Fig. 4:**
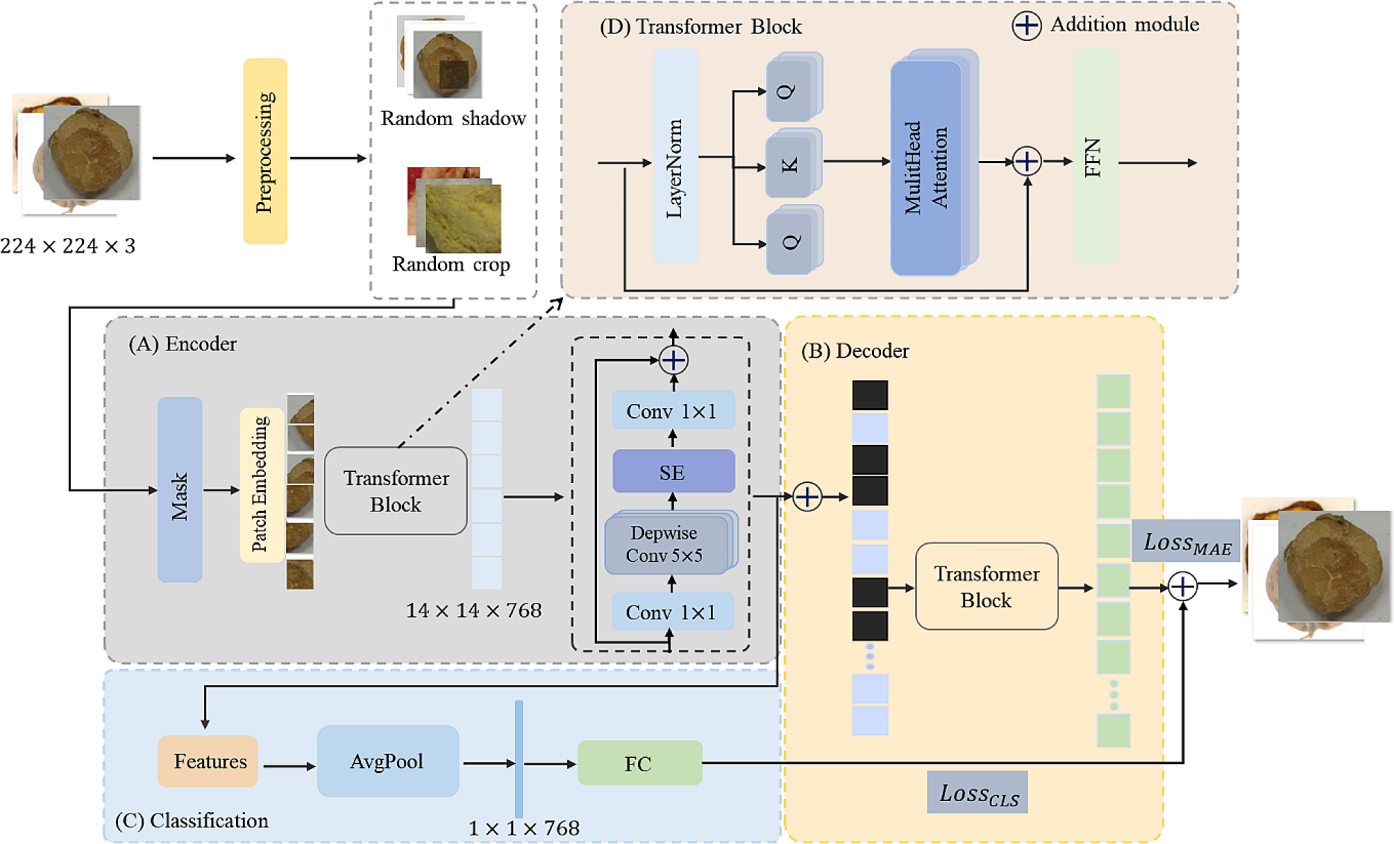
The overview of our identification model

We explain the different morphologies and color changes in our dataset. According to the properties of images, all data are detected to remove redundant pixels that contain no information. During the data collection processing, we collect multiple images of the same plant sample from different angles to enrich the diversity of data. Thus, we compile the specific quantity of each medicinal plant, and the distribution of the original dataset is shown in Fig. 4. The blue represents the raw samples, while the orange is the collected original data.

## Methodology

### Overview architecture

Our framework for CMP classification is shown in Fig. 1. Our model has 3 parts: (A) Encoder, (B) Decoder, and (C) Classification. Specifically, we use ViT to extract global features from different images. Additionally, we use MBConv to reduce the number of parameters and improve learning ability. Thus, the encoder is dedicated to learning the structural knowledge of images by incorporating MBConv and ViT. The patches and masks are processed to reconstruct the original images. Additionally, it harnesses the potential of the ViT in capturing essential information. Furthermore, a parallel supervised classification branch is introduced to make up the integration of global features within MAE. Lastly, the decoder aims to predict the features of the masked regions. As a result, the model accomplishes image classification.

Taking advantage of the sparsity of images and the learning ability of MAE, the combination Transformer with MBConv is used to extract local deep features. the loss is designed to compute for all patches. Moreover, we can generate diverse data by random masking, which provides a powerful regularization effect in supervised pre-training.

### Random data enhancement

**Fig. 5:**
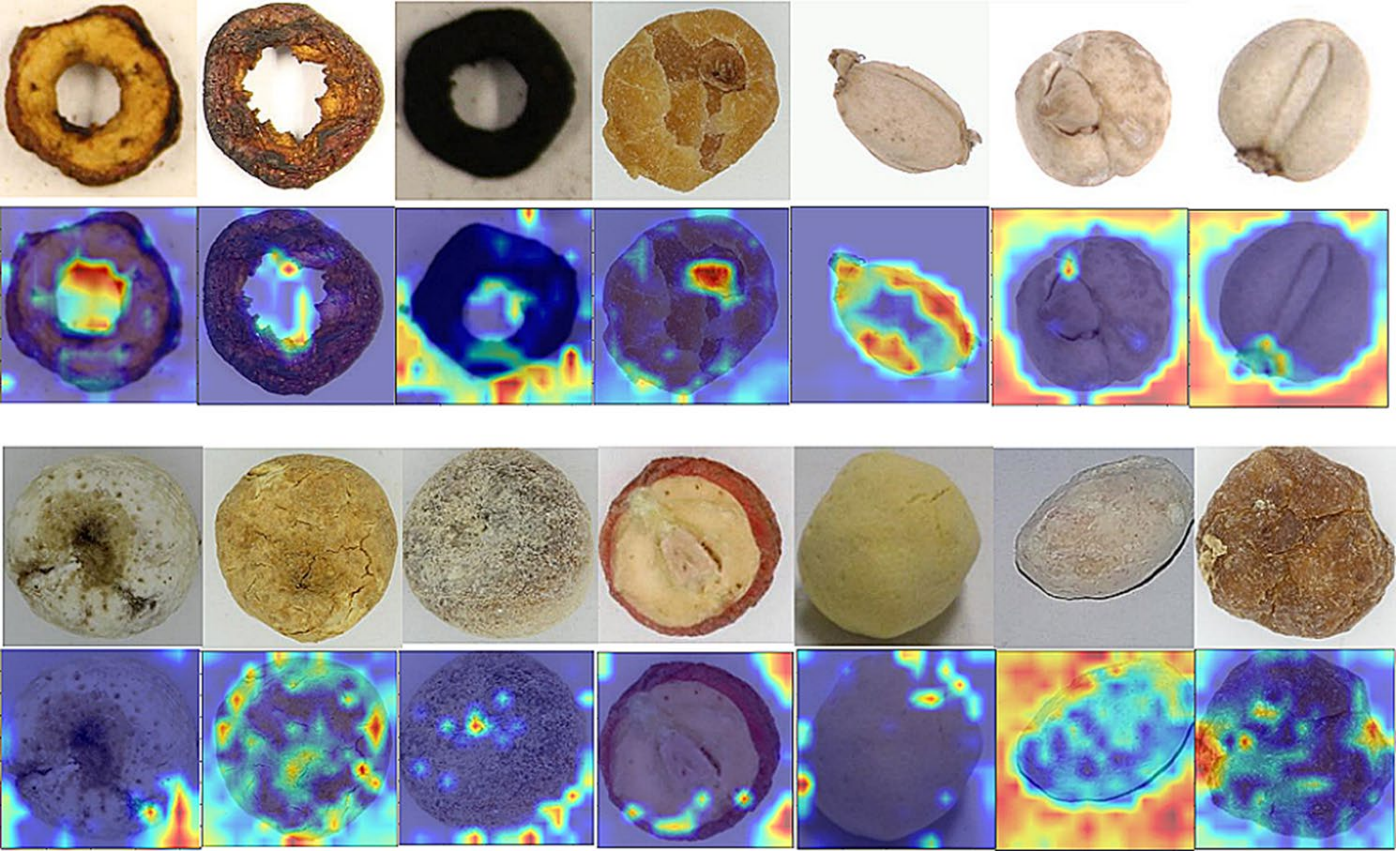
The Grad-CAM heatmap is based on MAE. The first row and Third row display original images, while the second row and 4-th row show the Grad-CAM heatmap results. The heatmaps are where the model is focused on

We first use Grad-CAM [38] to analyze which parts are more important for our model, the heatmap is illustrated in Fig. 5. Through the heatmap we can observe that our model focuses more on image edges, with limited attention to other areas. According to this observation, we propose a random data enhancement method that aims to improve the feature representation by selectively augmenting underrepresented minority images through random cropping and random shadowing.

#### Random shadow augmentation

**Fig. 6:**
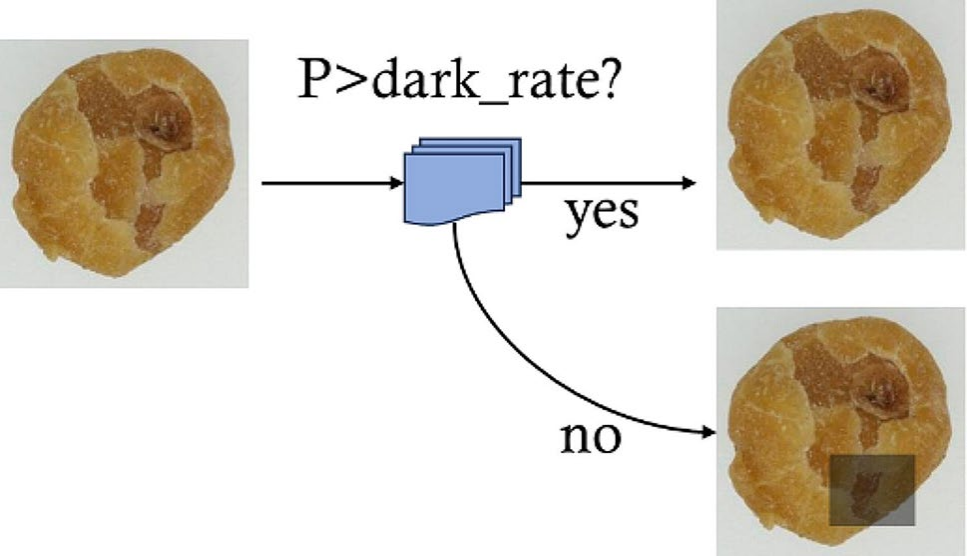
In the processing of Random shadow enhancement, $p$ is a random value between 0 to 1, $dark\_rate$ is the added shadow probability

As shown in Fig. 6, when processing the input image, a random value $p$ is generated within the range 0 to 1. If $p$ is less than $dark\_rate$, a random rectangular region is selected, and the values of RGB channels are decreased to create a shadow. Otherwise, the original image is kept.

The shadow areas ${D}_{rect}$ are computed in:

1 $$\begin{array}{c}X\left(i,j,c\right)= x\left(\text{i},\text{j},\text{c}\right)-shadow,\left(\text{i},\text{j}\right)\in {D}_{rect},c\in \left(\text{0,1},2\right)\end{array}$$

Where $x\left(\text{i},\text{j},\text{c}\right)$ represents the RGB channel in the area, $\text{s}\text{h}\text{a}\text{d}\text{o}\text{w},\left(\text{i},\text{j}\right)$is the levels of shadow intensity. $X\left(i,j,c\right)$ is the RGB value after shadow darkening.

#### Random crop augmentation

**Fig. 7:**
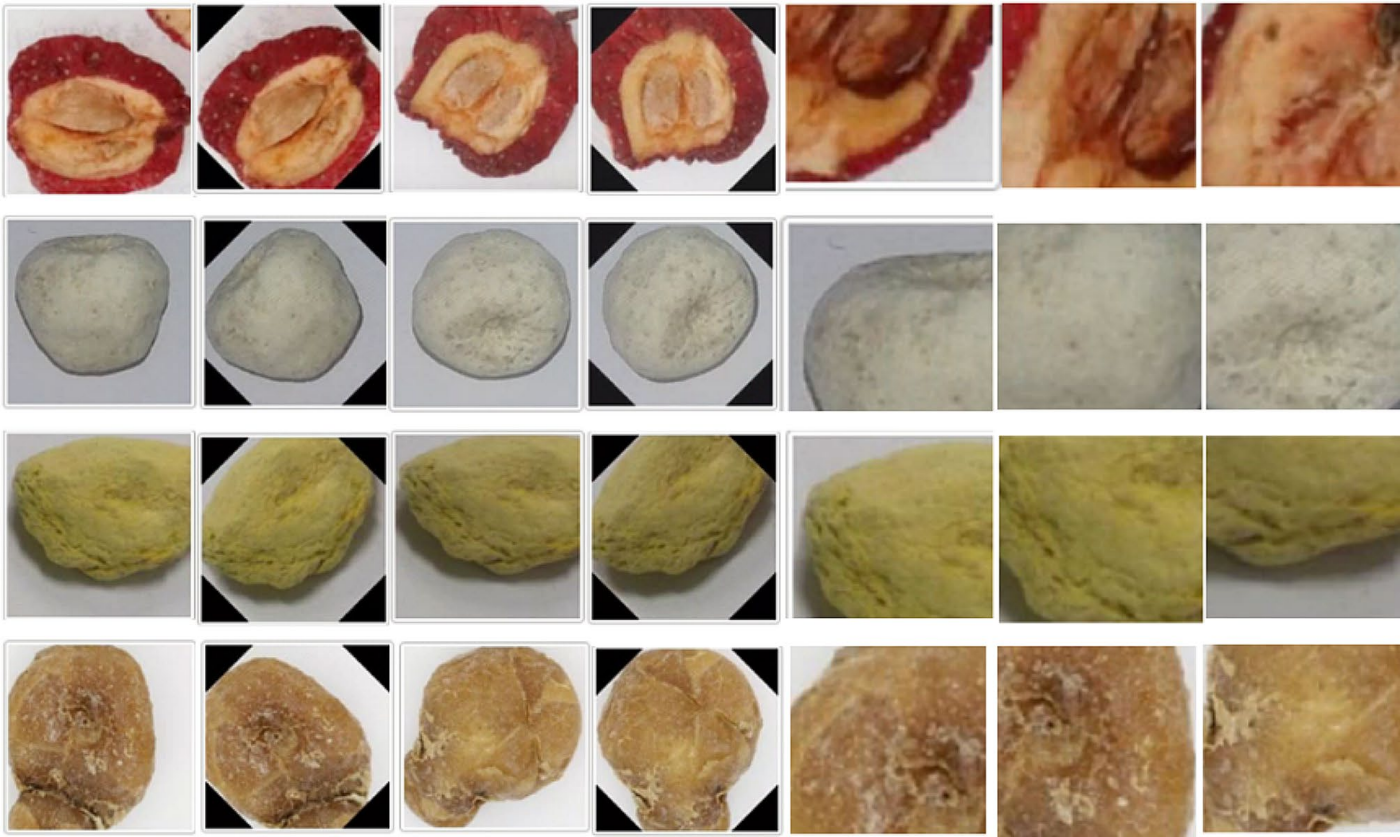
In the partial results of data augmentation results, each row shows the randomly cropped data of different classes, namely *shanzha*, *qingbanxia*, *jingbanxia*, and *jiangbanxia*, respectively

Simultaneously, a random local enhancement method is used for data preprocessing in this study. For the different classes, the proportion $\text{A}$ is calculated, and $1-\text{A}$ is used as the threshold. A random point and a random length are selected, and the local region is cropped. This is calculated in Formula 2.

2 $$\left\{ {\eqalign{ {\gamma = 1 + (1 - {\rm{A)}} \times d} \cr {\gamma = 1 - {\rm{(1}} - {\rm{A)}} \times d} } } \right.$$

where $d$ represents the Euclidean distance from the center, $d\in [0, 112]$. The threshold for random cropping is higher for fewer classes to enhance the capture of local information. Moreover, images are enhanced by random rotation and flip. The results of data augmentation are shown in Fig. 7.

### Nonlinear transform of self-attention

Generally, the image is denoted as $X\in {\mathbb{R}}^{h\times w\times C}$, which are divided into $N=h\times w/{P}^{2}$ non-overlapping patches.

3 $$X=\left\{{x}^{1},{x}^{2}\dots {x}^{n}\right\}$$

where ${x}^{n}\in {\mathbb{R}}^{{P}^{2}C}$ is the vector of patch, $P$ represents the resolution of patch. Each patch is projected as a 1D token embedding. Then, ${N}_{m}$ patches are randomly masked, and remaining ${N}_{v}$ are visible patches, ${N=N}_{m}+{N}_{v}$. ${X}_{v}=\left\{\left.{x}^{k}\right|k\notin M\right\}$ is defined as the set of visible pixels, ${X}_{m}=\left\{\left.{x}^{k}\right|k\in M\right\}$ is the set of masked pixels, where $M$ represents the indices of randomly masked pixels. Thus,

4 $$X={X}_{m}\cup {X}_{v}, {X}_{m}\cap {X}_{v}=\varnothing$$

In this study, the size of $224\times 224$ image is divided into $14\times 14$ grid of blocks, where each block has a size of $16\times 16$. Each visible patch is projected into an embedding, and the positional embedding${E}_{pos}$ is added to ensure the position of patch.

5 $$z=\left[{x}_{cls},{x}_{p}^{1}E,{x}_{p}^{2}E\dots .,{x}_{p}^{N}E\right]+{E}_{pos}$$

Then, it is computed by self-attention, the scaled dot-product attention is to obtain $Z\in {\mathbb{R}}^{d\times d}$.

6 $$Z=Attn\left(z\right)=Softmax(Q{K}^{\text{{\rm T}}}/\sqrt{w})V$$

the $Softmax$ attention $Attn(\cdot)$ with a global receptive field works as the following nonlinear mapping:

7 $$y{\prime }=LN(Z+FFN\left(LN\left(Z\right)\right))$$

where $LN(\cdot)$ is the Layer Normalization that essentially is a learnable column scaling with a shift, and $FFN(\cdot)$ is a standard two-layer feedforward neural network applied to the embedding of each patch. The scaled dot-product attention (6) of $Z$, the *jth* element of its *ith* row zi is obtained in Formula 8.

8 $${Z}_{i}^{j}=\frac{{e}^{{(Q{K}^{\text{{\rm T}}}/\sqrt{w})}_{i}}}{\sum _{j=1}^{h}{e}^{{(Q{K}^{\text{{\rm T}}}/\sqrt{w})}_{ij}}}. V= Softmax({q}_{i}{K}^{\text{{\rm T}}}/\sqrt{w})V$$

From Formula 7, the representation space for an encoder layer in MAE is spanned by the row space of V and is being nonlinearly updated layer-wise. The embedding for each patch serves as a basis to form the representation space for the current attention block.

Compared with CNN, the global self-attention mechanism ignores some local information about images, especially fine-grained features. Thus, ${y}^{{\prime }}$ is processed by depth-wise convolution to obtain deep details,

9 $$y=DepthConv\left({y}^{{\prime }}\right)$$

CNN is acting on a pixel level and is locally supported, thus having a small receptive field. MAE is globally supported, which means it can learn effectively the interaction between far-away patches. Transformer can aggregate coarse-grained features and expand the field of the convolutional blocks. Therefore, the hybrid structure exhibits superior performance.

### Supervised branch

The mask token is a learnable vector shared by masked patch, and then is connected to the unshuffled representation of the unmasked patches. Let ${N}_{m}\in {\mathbb{R}}^{1\times 1\times d}$ be the learned mask token embedding, and the index set of masked and unmasked patches as W and U, respectively. Thus, the affine maps are generated for $\left\{{Q}^{{\prime }}\right.,{K}^{{\prime }},\left.V{\prime }\right\}$.

10 $$\eqalign{ & \left\| {\mathop \sum \limits_{j = 1}^n Attn\left( {{Q_i},{K_j}} \right){V_i} - \mathop \sum \limits_{j \in U} \left( {Q{'_i},K{'_j}} \right)V{'_i}} \right\| \cr & \quad < {C_{{n^{ - 1}}}} \cr}$$

where $Attn(\cdot)$ denotes the attention kernel, which maps each patch’s embedding represented by the rows of Q, K to a measure of how they interact. It shows that the network interpolates the representation using global information from the embeddings learned by the MAE encoder, not just the nearby patches. For the embedding of masked patch $i\in \text{W}$, ${v}_{i}^{t+1}$ is the output embedding of a decoder layer, ${v}_{i}^{t}$ is the input from the encoder, then ${v}_{i}^{t+1}$ is computed:

11 $${v}_{i}^{t+1}=\sum _{j\in U}{a}_{j}{v}_{i}^{t}$$

Where ${a}_{j}({v}_{{i}_{1}}\dots .{v}_{{i}_{k}})$ is a set of weights based on unmasked patches, $U=\left\{{i}_{1}\dots {i}_{k}\right\}$. To prove that the latent representations of the masked patches are interpolated globally based on an inter-patch topology that is learned by the attention mechanism. To better learn the feature representations of data, the supervised label information is added. Simultaneously, we introduce a regularization term through the supervised branch to help prevent the model from overfitting to imbalanced data and improve its generalization ability.

### Loss functions

We optimize the reconstruction loss and classification loss at the same time. Reconstruction loss quantifies the disparity between the input data and the model’s reconstructed output. It incentivizes the model to acquire meaningful representations of the input data by penalizing inconsistencies between the original input and the reconstructed output. Classification loss is used to quantify the disparity between the predicted labels and the ground truth labels. The goal of the classification loss is to prevent the model from overfitting to imbalanced data and improve the generalization ability. The overall loss is shown:

12 $$Loss={Loss}_{MSE}+{Loss}_{ClS}$$

13 $${Loss}_{MSE}=\frac{1}{M\sum _{0}^{m}{\left(y-x\right)}^{2}}$$

According to the characteristics of the dataset, LabelSmooth [38] is selected as the classification loss function:

14 $${Loss}_{LS}=-\sum _{i}^{n}y\left(i\right)\text{log}\left(p\left({x}_{i}\right)\right)$$

15 $$y\left( i \right) = \left\{ {\matrix{ {{\varepsilon \over n}\,i \ne target} \cr {1 - \varepsilon + {\varepsilon \over n}\,i = target} \cr } } \right.$$

The penalty factor ε is introduced to emphasize the importance of low probability distributions. Therefore, it is used to address overfitting and insufficient supervision, and ε is set to 0.25.

## Results and discussions

### Training paraments

In this study, the model is optimized by the AdamW [39] algorithm. The initial learning rate is 1e-3, and the learning rate decay strategy is StepLR [40]. The batch size is set to 32, the gamma is set to 0.1. The experiment is based on Pytorch1.8.1 and Python3.9. The model is trained with Nvidia 2080Ti, and with 11G GPU. The final pre-trained model is obtained when reaching 400 epochs. For the fine-tuning, the initial learning rate is set to 1e-3, and the learning rate decay strategy is Cosine Annealing. The input image size is $224\times 224$, the batch size is set to 32, and the final model is obtained when it reaches 200 epochs.

### Random data enhancement

#### Random shadow augmentation

To test suitable parameters for random shadow augmentation, the experiments are performed. The four different shadow sizes (16, 32, 64, 128), three levels of shadow intensity (20, 30, 40), and four different dark rates (0.1, 0.2, 0.3, 0.4) are respectively selected. In fairness, the remaining parameters remain unchanged. The experimental results are shown in Table 1.

**Table 1:** Random shadow augmentation experiment

Shadow Sizes	Shadow Intensity	Dark_rate	Top-1 Accuracy (%)
16	30	0.4	98.19
32	30	0.4	98.19
64	30	0.4	97.41
128	30	0.4	96.87
32	30	0.3	**98.73**
32	30	0.2	98.19
32	30	0.1	98.24
32	20	0.3	98.19
32	40	0.3	98.1

The experimental results reveal that the excessively large shadow size and low brightness have a detrimental impact on the performance of the model. Further analysis reveals that only a portion of the data is affected by shadows. When we give a higher dark rate, we can see most of the training data becomes shadow-affected, resulting in excessive shadow processing. Conversely, the testing set contains fewer shadow-affected data, leading to a decrease in accuracy. The optimal results are attained with a shadow size of 32, a shadow intensity of 30, and a dark rate of 0.3. Simultaneously, 1000 data is added to each class.

#### Random crop augmentation

Similarly, to test suitable parameters for random crop augmentation, the experiments are performed. And the four different crop sizes (16, 32, 64, 128) are selected. The experimental results are shown in Table 2.

**Table 2:** Random crop augmentation experiment

Crop Sizes	Top-1 Accuracy (%)
16	96.67
32	96.97
64	97.78
128	**98.73**

The experimental results show that the small crop sizes can reduce the identification performance of the model. Upon further analysis, the limited features are learned by the small crop sizes. And the optimal results are attained with a crop size of 128. According to the size of the original data of each class, the data of random crop augmentation are listed in Table 3.

**Table 3:** The data for random crop augmentation

Classes	Number	Classes	Number
(A) *chaoshanzha*	905	(H) *shengbanxia*	1236
(B) *jiaoshanzha*	941	(I) *fabanxia*	1437
(C) *shanzhatan*	894	(J) *qingbanxia*	1342
(D) *jiangbanxia*	1275	(K) *shanzha*	1648
(E) *lubeimu*	774	(L) *jingbanxia*	1764
(F) *qingbeimu*	730	(M) *shuibanxia*	1403
(G) *chuanbeimu*	489	(N) *jiangnanxing*	1342

### Evaluation of identification performance

We split the data into 3 parts, that is, 70% of the data as the training set, 15% of the data as the testing set, and the remaining 15% of the data as the verification set. To measure our model the identification accuracy, we select 4 metrics to measure our model performance, including, Precision, Recall, Specificity, and F1 Score [41, 42].

16 $$\text{P}\text{r}\text{e}\text{c}\text{i}\text{s}\text{i}\text{o}\text{n}=\frac{TP}{TP+FP}$$

17 $$\text{R}\text{e}\text{c}\text{a}\text{l}\text{l}=\frac{TP}{TP+FN}$$

18 $$\text{S}\text{p}\text{e}\text{c}\text{i}\text{f}\text{i}\text{c}\text{i}\text{t}\text{y}=\frac{TN}{FP+TN}$$

19 $$\text{F}1 \text{S}\text{c}\text{o}\text{r}\text{e}=\frac{2\times (\text{P}\text{r}\text{e}\text{c}\text{i}\text{s}\text{i}\text{o}\text{n}\times \text{R}\text{e}\text{c}\text{a}\text{l}\text{l})}{\text{P}\text{r}\text{e}\text{c}\text{i}\text{s}\text{i}\text{o}\text{n}+\text{R}\text{e}\text{c}\text{a}\text{l}\text{l}}$$

where TN is the number of True Negative, and TP is the number of True Positive. FN indicates the number of False Negative, and FP indicates the number of False Positive. The detailed results are shown in Table 4. Our method achieves satisfactory results in these 4 metrics across different classes.

**Table 4:** The Experimental classification results

Classes	Precision	Recall	Specificity	F1 Score
(A) *chaoshanzha*	1.0	1.0	1.0	1.0
(B) *jiaoshanzha*	1.0	1.0	1.0	1.0
(C) *shanzhatan*	1.0	1.0	1.0	1.0
(D) *jiangbanxia*	1.0	0.988	1.0	0.994
(E) *lubeimu*	0.99	1.0	1.0	0.995
(F) *qingbeimu*	0.995	1.0	1.0	0.997
(G) *chuanbeimu*	0.999	1.0	1.0	0.999
(H) *shengbanxia*	0.998	0.998	1.0	0.998
(I) *fabanxia*	0.96	0.957	0.994	0.958
(J) *qingbanxia*	0.936	0.938	0.986	0.937
(K) *shanzha*	0.971	0.972	0.992	0.971
(L) *jingbanxia*	1.0	0.985	1.0	0.992
(M) *shuibanxia*	0.979	1.0	1.0	0.993
(N) *jiangnanxing*	0.991	0.996	1.0	0.993

**Fig. 8:**
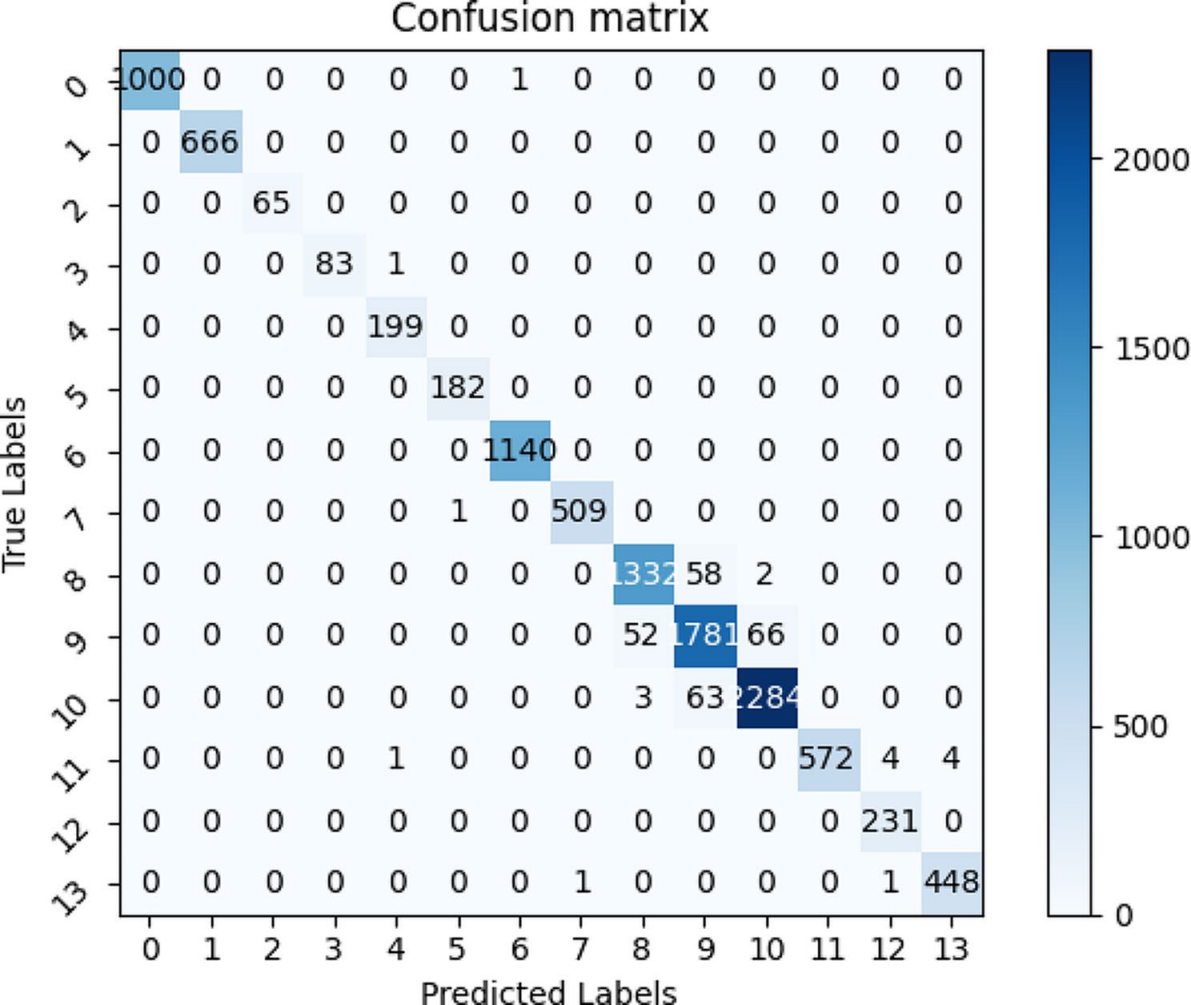
The experimental results of the confusion matrix. The numbers from 0 to 13 correspond to different classes. The columns represent the predicted labels, the rows represent the true labels. The values corresponding to rows and columns have indicated the number of correct classes predicted from true data

**Fig. 9:**
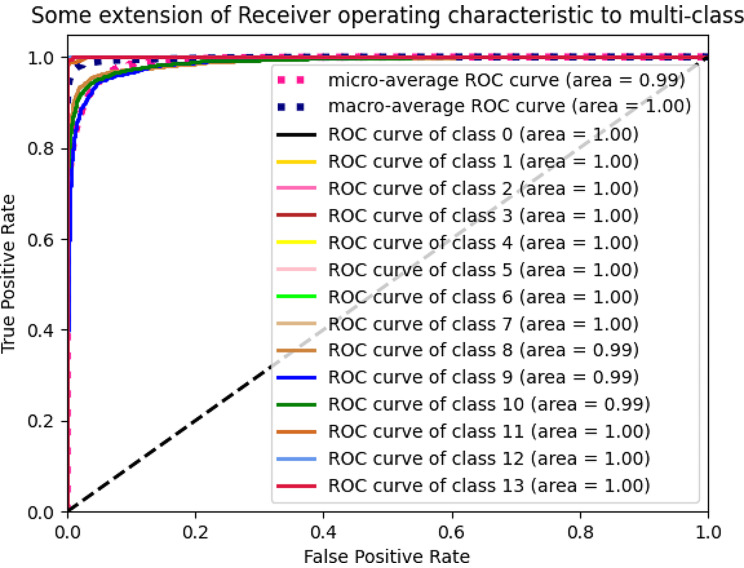
The experimental results of Receiver Operating Characteristic (ROC). The number from 0 to 13 corresponds to different classes. Based on the confusion matrix, ROC is computed to reflect the difference between the True Positive Rate and False Positive Rate. The range of ROC curve is between 0 and 1 (1 is best, 0 is lowest)

Our model achieves excellent results. Additionally, to further analyze our model performance, we visualize the confusion matrix and ROC curve, as shown in Figs. 8 and 9, respectively.

The results are harmonious with the classification results in Table 4. There are certain errors among different classes, especially *qingbanxia* and *jingbanxia*. *qingbanxia* and *jingbanxia* are both processed from *banxia* by different processing methods, resulting in similar morphology and textures. And the color is the most prominent distinction. Consequently, variations in angles and lighting conditions can impact visual differentiation.

### Comparison with different models

Multiple different ConvNets and state-of-the-art Transformer models are compared with ours, to verify the significance of the proposed method. Focalloss has been chosen as the loss function for all, including VGG [43], ResNet [44], DenseNet [45], EfficientNet [46], etc. Otherwise, to reflect the significant effect on the computation cost, the frame per second (FPS) and floating-point operations per second (FLOPs) are computed. The comparative experimental results are shown in Table 5.

**Table 5:** The Experimental classification results

Method	Top-1 Accuracy (%)	AUC (%)	FPS	FLOPs
VGG16 [43]	95.74	99.0	30.056	248.11
ResNet50 [44]	96.46	100	46.694	65.75
MobileNetsV2 [45]	94.96	98.0	41.442	5.01
DenseNet169 [47]	96.62	100	32.357	54.34
EffcientNet-B0 [46]	96.57	100	33.126	0.22
ViT [37]	93.96	98.0	10.607	299.49
CoAtNet [48]	93.58	98.0	36.638	43.81
MAE [35]	96.64	100	11.08	301.35
Ours	**98.73**	**100**	**11.176**	**316.53**

**Fig. 10:**
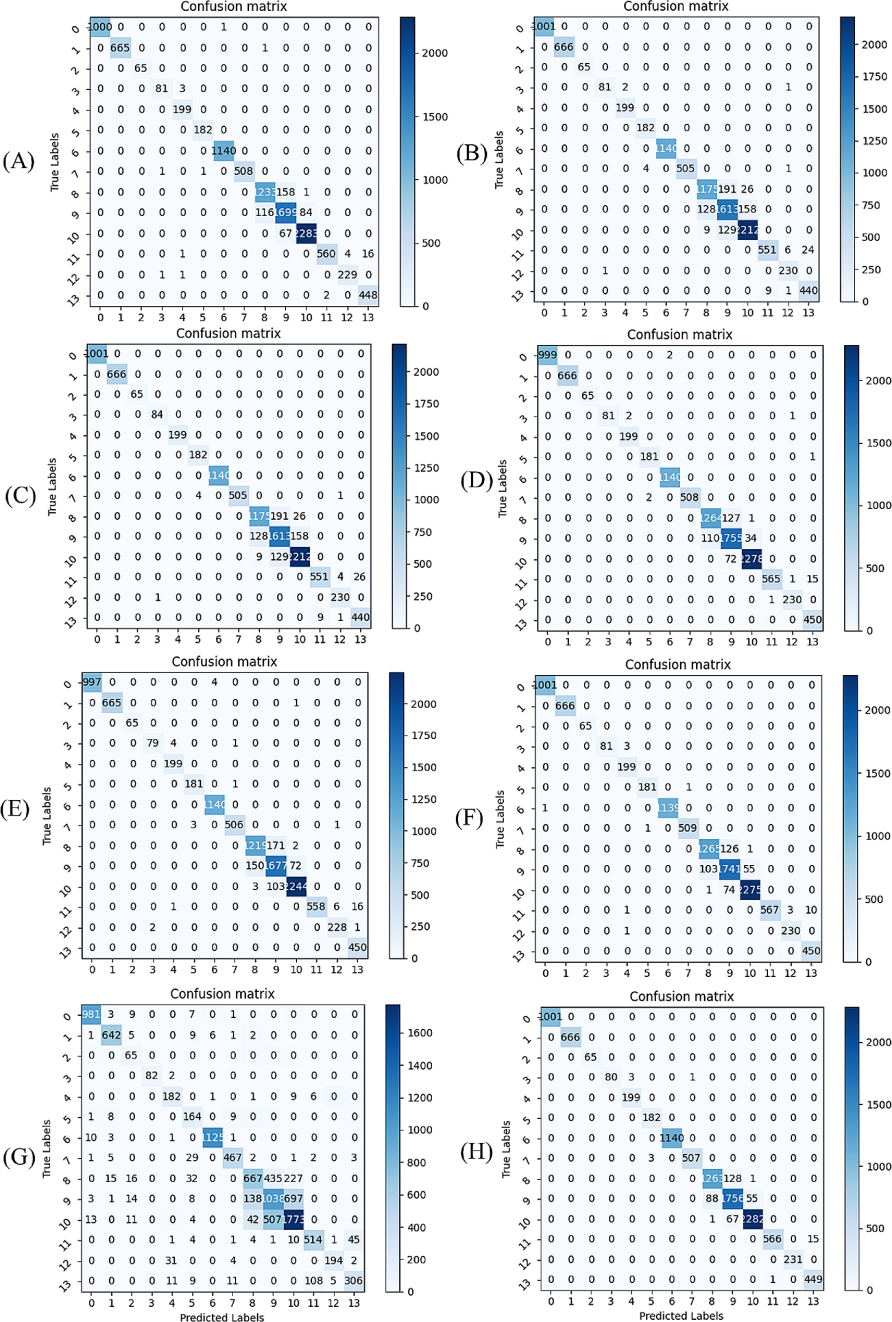
The experimental results of a confusion matrix for different models. (**A**) VGG (**B**) CoAtNet (**C**) DenseNet (**D**) EffcientNet (**E**) MobileNets (**F**) ResNet (**G**) ViT (**H**) MAE

As shown in Table 5, the proposed method has achieved the highest Top-1 accuracy, while CoAtNet had the lowest Top-1 classification accuracy of 93.58%. Compared to MAE, ours improved by 2.09%. Notably, CoAtNet displayed constraints in its feature-capturing capabilities, and ViT necessitated larger datasets by Transformer modules. The discriminative efficacy of these two models falls short in comparison to the others. Ours exhibits a higher FPS compared to the MAE, demonstrating the small computation cost. Compared with ViT and CNN models, ours has a lower FPS speed due to its increased computational demands. ViT typically requires more computational resources to process input images, including patch segmentation, patch embedding, and multi-layer Transformer modules. In contrast, CNN models leverage features such as local connections and parameter sharing, leading to higher computational efficiency during image processing. Additionally, ViT often necessitates longer training times and a greater number of parameters to achieve optimal performance, which consequently results in slower inference speeds. The experimental results of the confusion matrix for different models are shown in Fig. 10.

### Analysis of experimental results

#### Different modules comparison

We conduct an ablation experiment to prove the availability of our model, that is, we compare the model performance by using different modules. For a fair comparison, we keep the remaining parameters and settings unchanged. The comparative results are shown in Table 6.

**Table 6:** The identification results of different ablation experiments

Method	ImageNet pretrained	Top-1 Accuracy (%)	AUC (%)
MAE [35]	-	93.1	98.0
MAE [35]	√	96.64	100
MAE [35] + Conv	-	92.50	98.0
MAE [35] + Conv	√	93.73	98.0
MAE [35] + Depthwise Conv [36]	-	96.98	100
MAE [35] + Depthwise Conv [36]	√	97.79	100
MAE [35] + CLS branch	√	98.33	100
Ours	√	**98.73**	**100**

The experimental results reveal that introducing convolution layers prior to the network leads to an enlarged receptive field surpassing the dimensions of the masked patches. Consequently, information leakage occurred, leading to a decrease in classification accuracy. Furthermore, it can be observed that the introduced classification branches can lead to a 1.69% improvement over MAE. During training, the classification loss is added to compute for all labels, not just the masked labels. Supervised learning can enhance the integration of global features, and the ability to learn local-global features is strengthened. Additionally, the ablation experiment results demonstrate significant improvements achieved through pre-training weight.

#### Visualization of different models

**Fig. 11:**
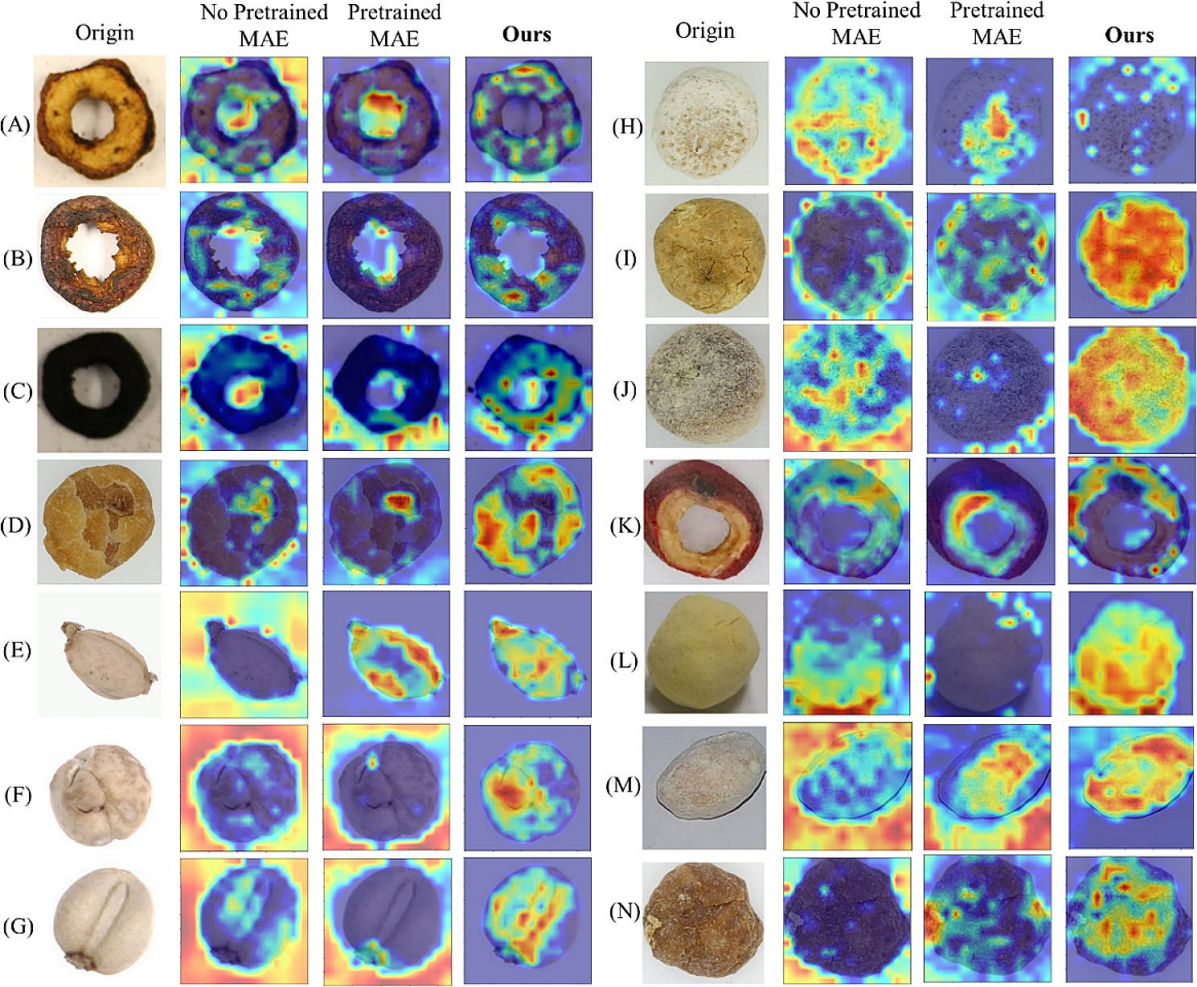
The visualization of the different models for original data. The highlighted areas of the CAM heatmap represent the model considered most relevant to each class. The heat maps of each class are randomly selected. The first is the original image, the second is the no-pretrained MAE, the third is the pretrained MAE, and the last is ours

**Fig. 12:**
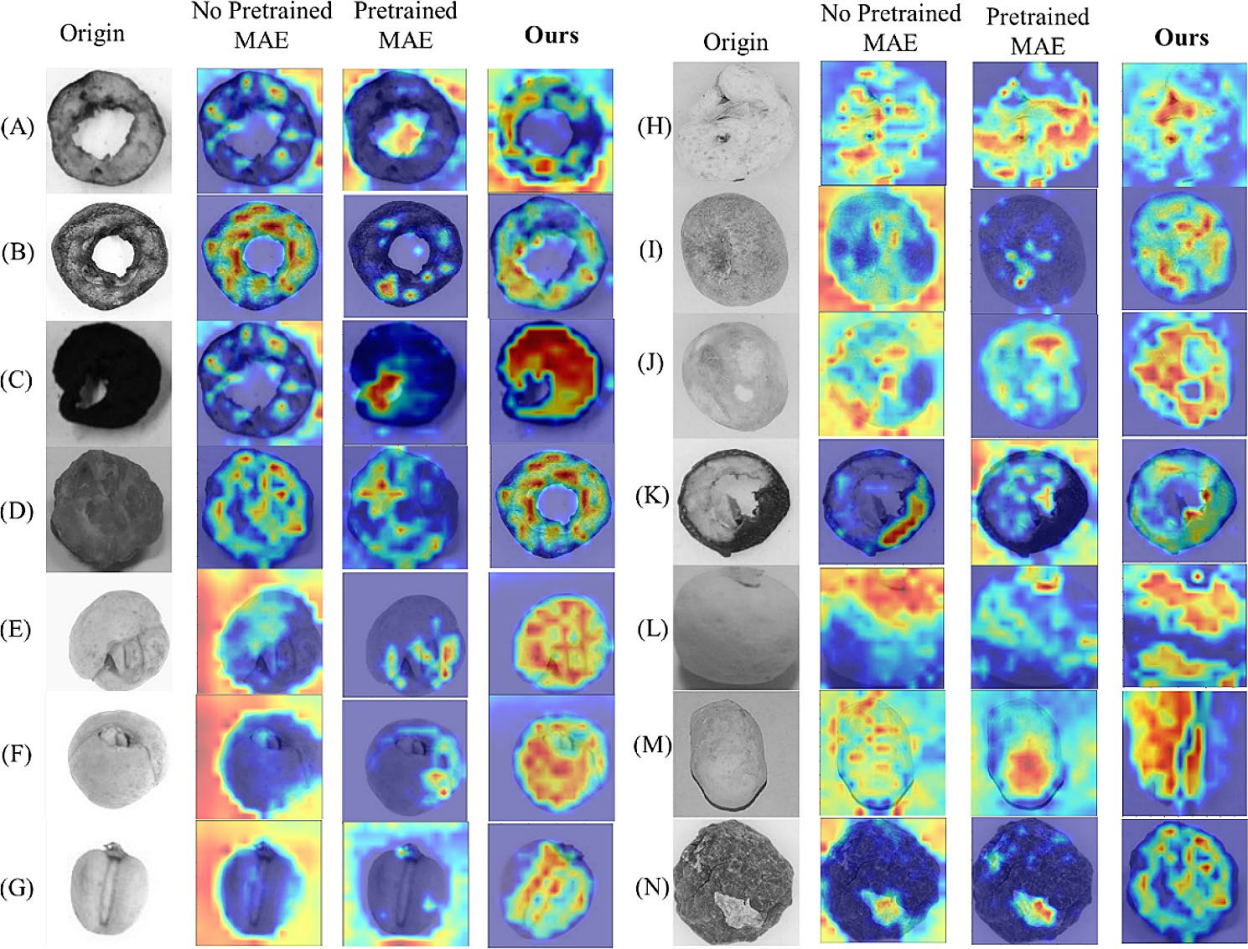
The visualization of the different models for different color backgrounds. The heat maps of each class are randomly selected

**Fig. 13:**
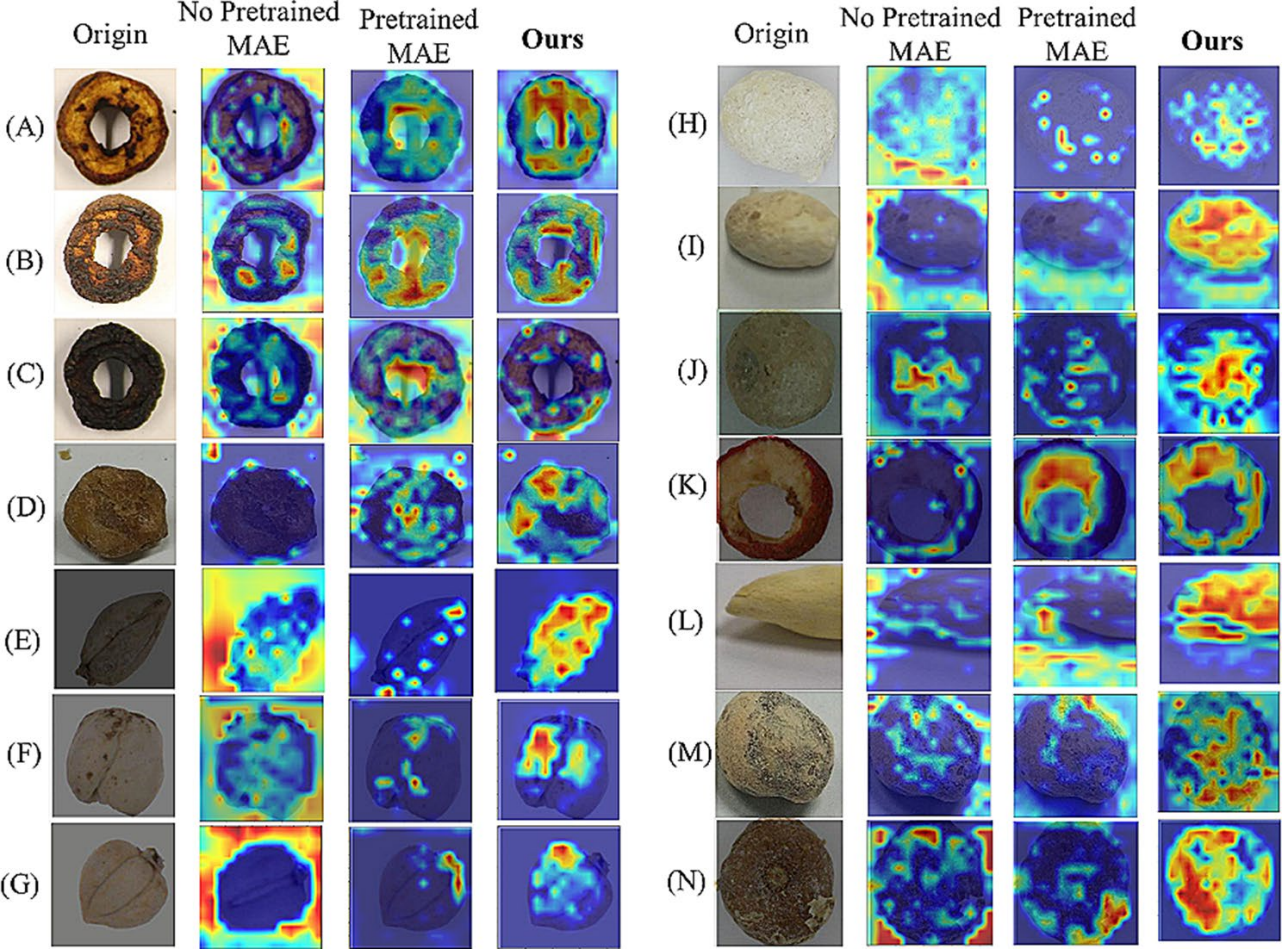
The visualization of the different models for different lighting and shadowing. The heat maps of each class are randomly selected

**Fig. 14:**
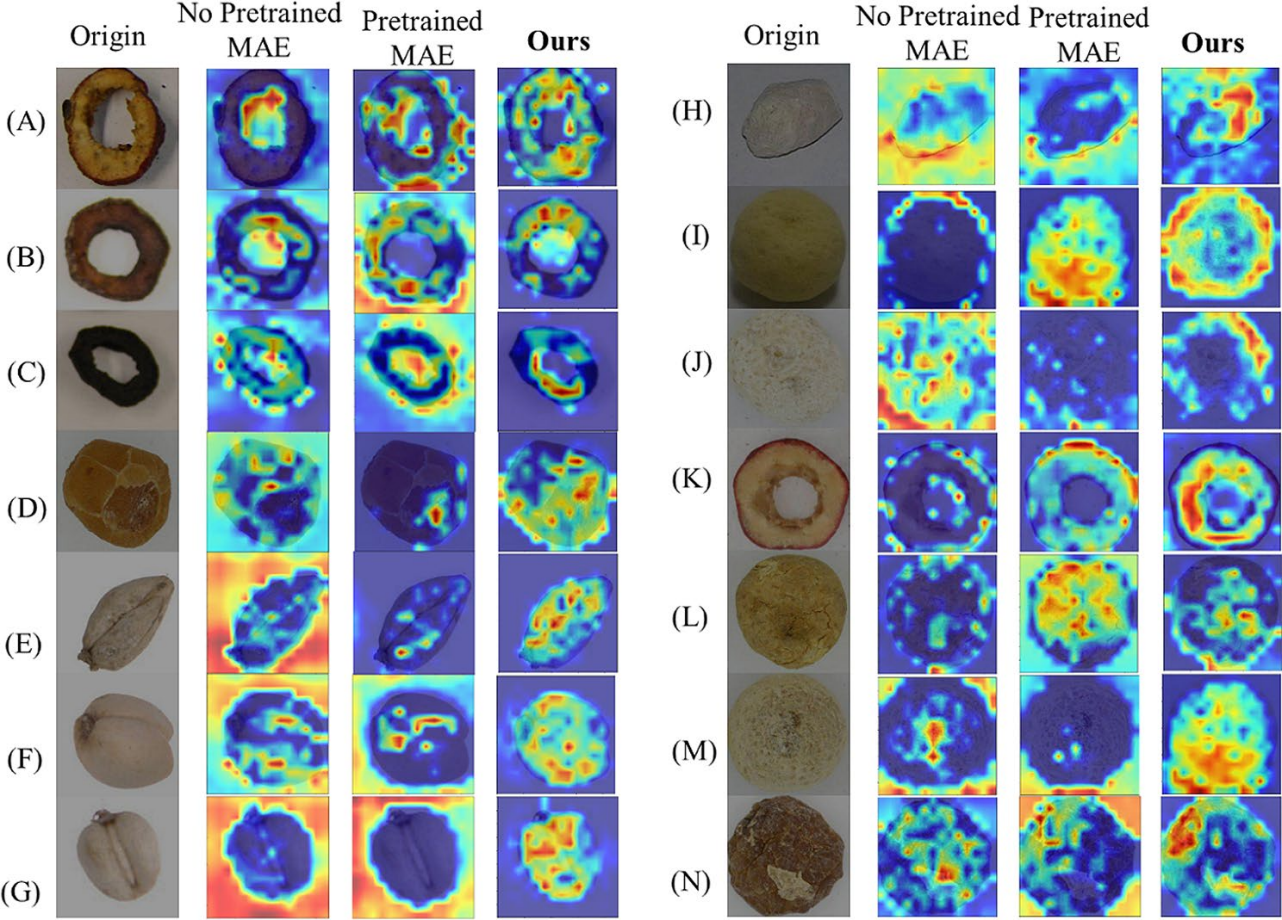
The visualization of the different models for different reflectance. The heat maps of each class are randomly selected

To illustrate the differences between the MAE and ours, we conduct another experiment, that is, visualize results by using a Grad-CAM heat map. Through the comprehensive analysis of the activation distribution in the feature maps, we can identify that our model is more focused on regions of the image. The heat maps of the original images are shown in Fig. 11. Meanwhile, to verify the influence of lighting and shadowing on results, we conduct other experiments, that is, we select some images that contain lighting difference and shadowing differences, the results are shown in Figs. 12 and 13, and Fig. 14.

The second column shows the feature maps that are obtained without using pre-trained weights from MAE. The third column displays feature maps by using MAE, in this case, MAE is fine-tuned through pre-trained weights from ImageNet. The fourth column is the heatmap for the proposed model in this paper. Figure 11. shows a comparison of heat maps for original images. Figure 12. is the schematic comparison of heat maps for different lightings. Figure 13. is the comparison of heat maps for different types of images under multiple models in the case of shadowing. Figure 14. is the comparison of the heat maps for various models under different reflectance and colors. Various methods exhibit diverse focal points within images. MAE tends to concentrate on less pertinent regions around the target, with restricted attention. Conversely, our approach uniquely centers on the target of images, encompassing a wider area and showcasing heightened intensity. Simultaneously, for the visualization of different color backgrounds, different lighting and shadowing, and different reflectance, our model still pays more significant attention to the target. Consequently, ours has higher accuracy. Furthermore, adopting the self-supervised Pretrained-Finetune training effectively boosts accuracy and reinforces the generalization of the model.

#### Comparison of different iterations

**Fig. 15:**
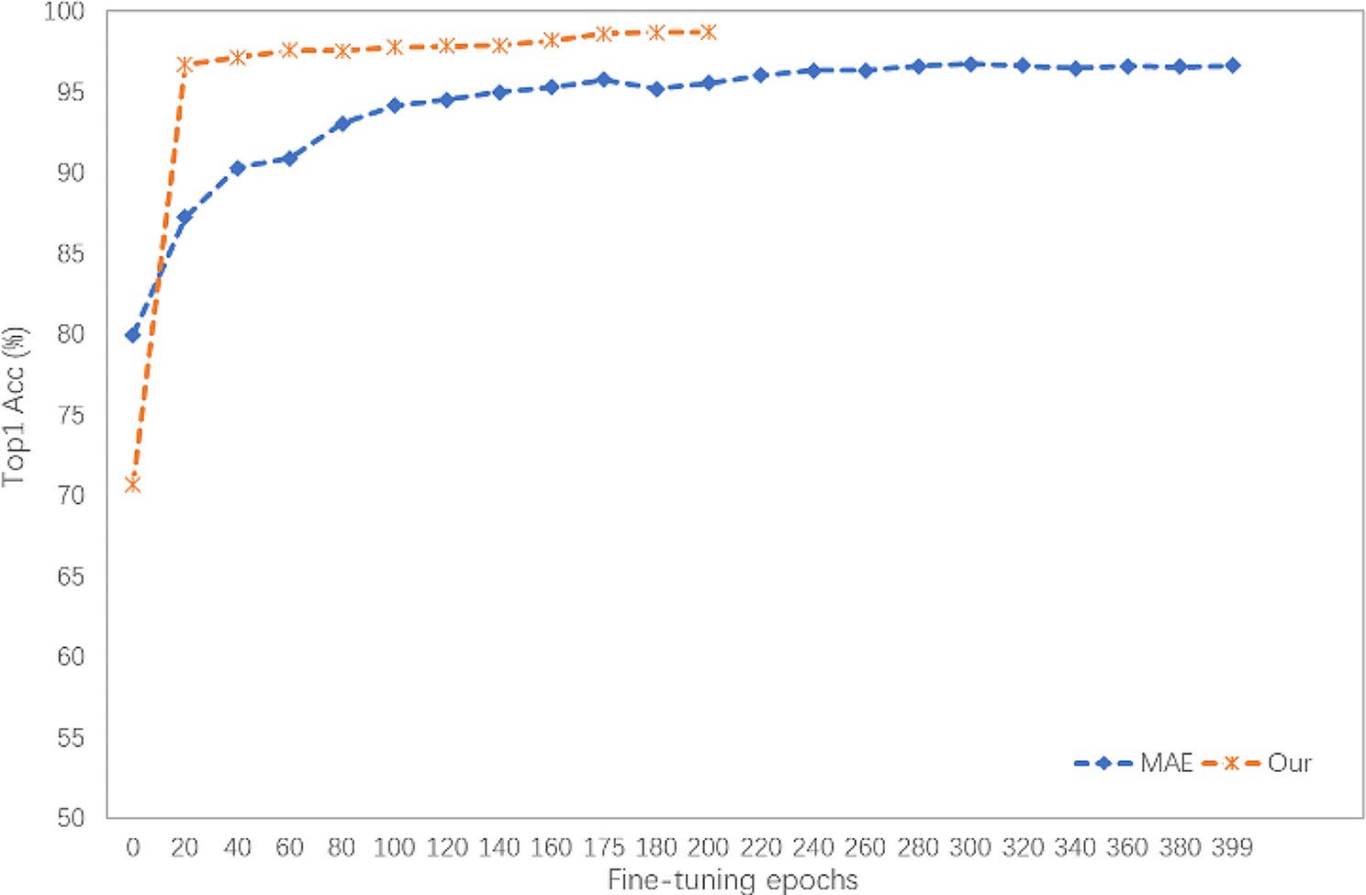
The experimental results of different iterations

To investigate the influence of different iterations. Thus, we examine the convergence of the model under different iterations. The experimental results are shown in Fig. 15.

Figure 15 points our model has a quicker convergence speed and achieves a higher accuracy of 98.73% by the 175th epoch. In comparison, MAE achieves a lower accuracy of 96.64% after 400 epochs. Furthermore, it attains an accuracy of 95.58%, when MAE reaches 200 epochs. Ours has included a supervised classification branch, making it relatively easier to saturate the pre-trained model. Additionally, our method encompasses all the hyperparameters of MAE while introducing additional branches, thereby contributing to enhanced convergence speed and training accuracy.

#### Different optimizer comparison

**Fig. 16:**
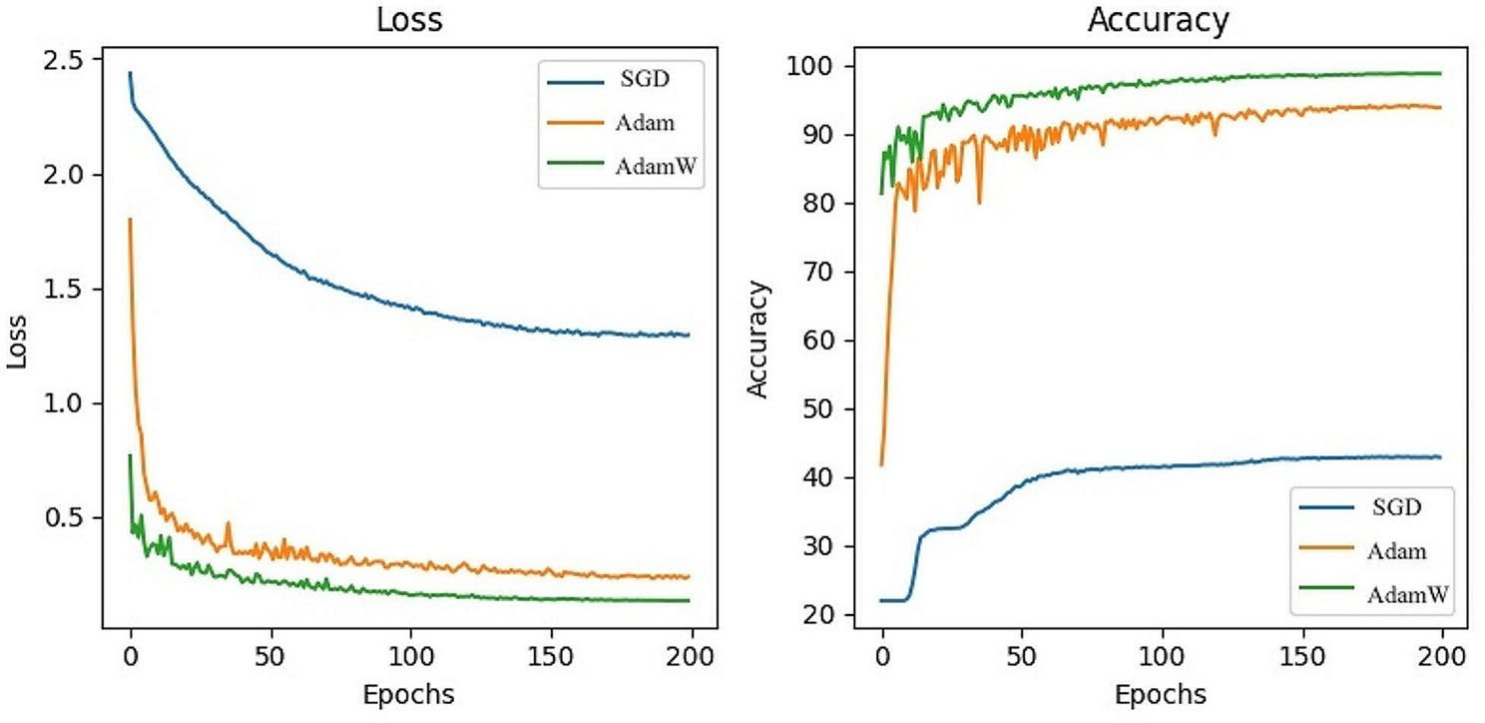
The comparison of experimental results of different iterations

Different optimization algorithms [49–53] may affect the speed of coverage, and leading model converges at different local minima. Following existing paper experiences, we select AdamW [39] as our model optimizer. To further explore the effect of optimizer, we conducted an experiment that used different optimizers, including Adam, and SGD. The comparison experimental results are shown in Fig. 16.

From the changes in loss and accuracy shown in Fig. 16, the convergence speed and the generalization performance of AdamW are significantly superior to the other two optimizers. AdamW introduces the concept of weight decay, which helps prevent overfitting by encouraging the model to utilize smaller parameter values. Consequently, it encourages better generalization of unseen data. Additionally, weight decay is decoupled from the parameter update process, thereby enhancing optimization stability and convergence.

#### Different parameter selections

We conduct comparative experiments by selecting different batch sizes and learning rates. By adjusting the values of input hyperparameters, we evaluate the influence of input parameters on the output parameters. The experimental results are shown in Tables 7 and 8.

**Table 7:** The identification results of different ablation experiments

batch sizes	Top-1 Accuracy (%)
8	98.92
16	98.73
32	98.73

**Table 8:** The identification results of different ablation experiments

learning rates	Top-1 Accuracy (%)
1e-3	98.19
1e-4	97.53
1e-5	96.28

Considering the GPU memory, the batch sizes are set to 8, 16, and 32 respectively. In fairness, the remaining parameters remain unchanged. From Table 7, it is interesting to see that when we set the batch size to 16 and 32, the accuracy is the same. However, when the batch size is set to 8, although the accuracy is the highest (with 0.2 surpassed), the training time is the longest. Therefore, to balance the relationship between training speed, generalization ability, and memory consumption, we ultimately choose a batch size of 32.

Similarly, to measure the impact of the learning rate, we select different learning rates such as 1e-3, 1e-4, and 1e-5, the results are shown in Table 8. As we can see, a small learning rate leads to slow convergence, thus resulting in the lowest accuracy at the same epoch. When we select a larger learning rate 1e-3, it has higher accuracy.

### Different datasets and performance trade-offs

#### Chinese medicinal blossom dataset

The blossom images of traditional Chinese medicinal herbs were captured by Google search. The images were divided into 12 categories, including (1) *syringa*, (2) *bombax malabarica*, (3) *michelia alba*, (4) *armeniaca mume*, (5) *albizia julibrissin*, (6) *pinus massoniana*, (7) *eriobotrya japonica*, (8) *styphnolobium japonicum*, (9) *prunus persica*, (10) *firmiana simplex*, (11) *ficus religiosa* and (12) *areca catechu*. The total number of images acquired is 12,538 [54]. The comparative results based on our model are shown in Table 9.

**Table 9:** The experimental classification results based on chinese medicinal blossom

Methods	Top-1 Accuracy (%)	AUC (%)
VGG16 [43]	84.47	94.0
ResNet50 [44]	89.87	95.0
MobileNetsV2 [45]	96.78	100
DenseNet169 [47]	93.85	98.0
EffcientNet-B0 [46]	97.02	100
ViT [37]	90.75	95.0
CoAtNet [48]	93.38	98.0
MAE [35]	96.89	100
Ours	**97.34**	**100**

From the quality comparison, we can see our method exhibits better classification accuracy when compared to MAE. And it maintains the highest accuracy than other mainstream methods.

#### Medicinal leaf dataset

This dataset comprises 30 different species of medicinal herbs including *Santalum album*, *Muntingia calabura*, *Plectranthus amboinicus*, *Brassica juncea*, etc [55]. Each species consists of 60 to 100 high-resolution images. The classification results obtained by our model are shown in Table 10.

**Table 10:** The Experimental classification results based on Medicinal Leaf

Methods	Top-1 Accuracy (%)	AUC (%)
VGG16 [43]	83.96	94.0
ResNet50 [44]	88.27	97.0
MobileNetsV2 [45]	98.4	100
DenseNet169 [47]	97.54	100
EffcientNet-B0 [46]	99.2	100
ViT [37]	93.72	98.0
CoAtNet [48]	97.38	100
MAE [35]	98.93	100
Ours	**99.47**	**100**

The results show that traditional convolutional neural networks which are traditional CNN methods have limited classification performance on this dataset. In contrast, our method demonstrates a clear advantage, surpassing MAE by 0.54%.

## Conclusion

CMPs are practiced and refined with a history of exceeding thousands of years for both health-protective affection and clinical treatment in China. However, the confusion by different processed conditions and cultivation environments affected clinical safety and medication efficacy are reported. The physicochemical and biological methods are high professional threshold and inefficient. Furthermore, manual-based identification methods are cumbersome and time-consuming. Thus, the visual feature-based approach is an increased interest in the advantages of being fast, accurate, and non-invasive. In this paper, a visual multi-varieties CMPs image dataset is constructed. Then, a random local data enhancement preprocessing method is proposed to enrich the feature representation for imbalanced data by random cropping and random shadowing. A novel hybrid supervised pre-training network is proposed to expand the integration of global features within MAE by incorporating a parallel classification branch. It can effectively enhance the feature capture capabilities by integrating global features and local details. Besides, the newly designed losses are proposed to strengthen the training efficiency and improve the learning capacity, based on reconstruction loss and classification loss. Extensive experiments are performed on our dataset as well as the public dataset. Experimental results demonstrate that our method has the best accuracy of 98.73%, which is superior to the state-of-the-art methods. Ours can transfer massive general knowledge to enhance feature capture capabilities, and to address the challenges of overfitting, end-to-end training difficulties in deep learning-CMPs. Moreover, it holds significant real-world applications value and benefits the development of accurate identification of medical plants.

## Data Availability

The original dataset in the study is released on GitHub (https://github.com/Tanchaoqun123/CHMs).
